# A Rare Cause of Heart Failure Treated by Heart Transplantation: Noncompaction of the Ventricular Myocardium

**DOI:** 10.1155/2009/725879

**Published:** 2010-03-18

**Authors:** Julien Bordes, Bertrand Jop, Sandrine Imbert, Sami Hraiech, Frédéric Collard, François Kerbaul

**Affiliations:** ^1^Department of Intensive Care, Sainte Anne Hospital, 83800 Toulon, France; ^2^Department of Cardiac Surgery, La Timone Hospital, 13000 Marseille, France; ^3^Department of Anesthesia and Intensive Care Unit, La Timone Hospital, 13000 Marseille, France

## Abstract

Noncompaction of the ventricular myocardium is a rare cardiomyopathy due to an arrest of myocardial morphogenesis. The characteristic echocardiographic findings are prominent myocardial trabeculations and deep intertrabecular spaces communicating with the left ventricular cavity. The clinical manifestations include heart failure (HF) signs, ventricular arrhythmias, and cardioembolic events. We describe an illustrative case of noncompaction of the ventricular myocardium associated with bicuspid aortic valve, a 42-year-old male presenting a refractory acute heart failure successfully treated by emergency heart transplantation.

## 1. Introduction

Noncompaction of the ventricular myocardium (NVM) is a recently described cardiomyopathy. It is an anomaly of myocardial morphogenesis that leads to persistent prominent ventricular trabeculations and deep intertrabecular recesses communicating with the ventricular cavity [1]. The prognosis is poor and heart transplantation has to be considered in patients with severe symptoms [2]. This disease is rare and may be undiagnosed. Herein we report a late presenting case of NVM with end stage congestive heart failure, successfully treated with heart transplantation.

## 2. Case Presentation

A 42-year-old man was referred in for evaluation of progressive dyspnea. He had a history of aortic bicuspid valve, with a poor medical followup. A transesophageal echocardiography demonstrated the aortic bicuspidy with grade III aortic insufficiency. The left ventricle was dilated with an ejection fraction of 40%. A surgical valve repair was proposed though the patient refused surgery. After 3 months, the patient was admitted to hospital emergently with congestive heart failure. Diuretic and dobutamine infusions were started, as well as amiodarone after an episode of ventricular fibrillation. Despite this medical treatment, his condition worsened and he was transferred to our cardiac surgical intensive care unit.

**Figure 1:**
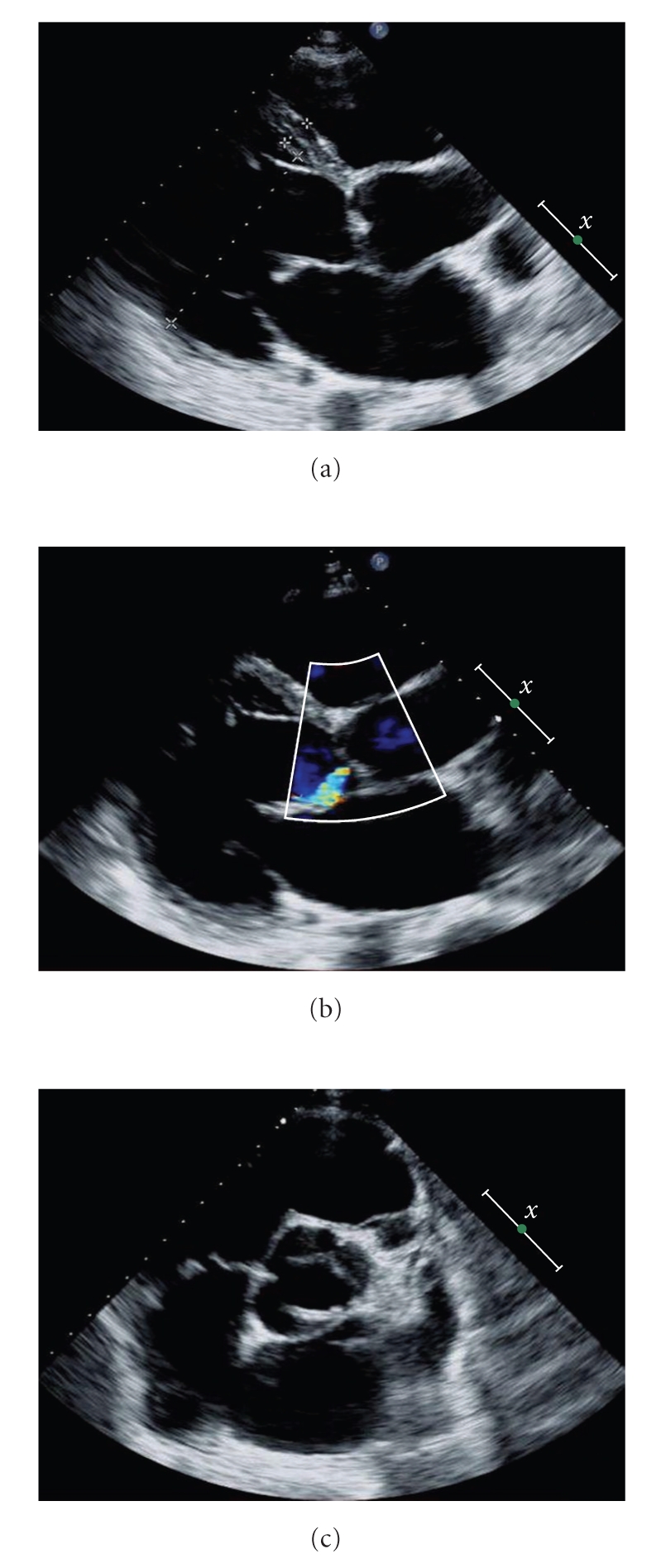
Transthoracic echocardiography. (a) Parasternal long-axis view, the left ventricule telediastolic diameter is 80 mm. (b) Parasternal long-axis view, aortic regurgitation. (c) Short-axis view at the level of the aorta, bicuspid aorti valve.

**Figure 2:**
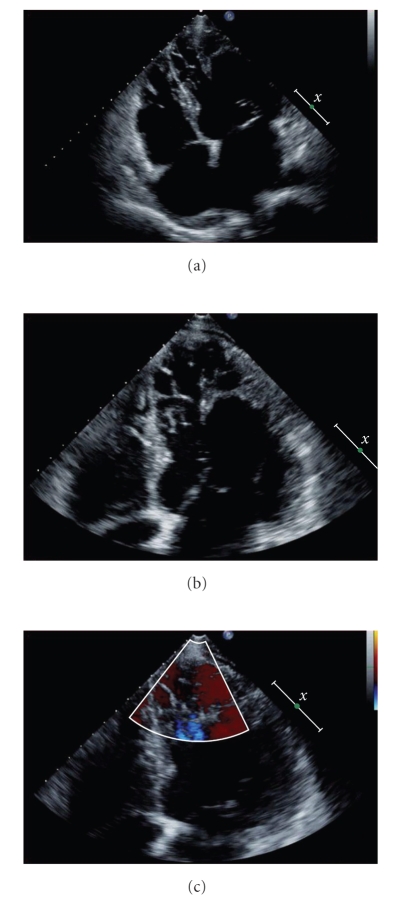
Transthoracic echocardiography. (a) and (b) Apical four-chamber view, characteristic, multiple, prominent muscular trabeculations and intertrabecular recesses. (c) Color Doppler mode, blood flow within the deep intertrabecular recesses.

On admission, the heart rate was 89/min and the arterial pressure was 80/50 mmHg on dobutamine 20 *μ*g/kg/min. The patient's oxygen saturation was 99% on room air. The transthoracic echocardiography showed severely depressed left ventricular systolic function with an ejection fraction of 25%. The left ventricle was dilated with a severe functional mitral regurgitation (Figure 1). Although previous echocardiography demonstrated significant aortic insuficiency, the aortic regurgitation was measured minimal (Figure 1). In addition, repeat echocardiography visualized prominent ventricular trabeculations in left ventricle, predominant to apical and mid-inferior areas (Figure 2). The maximal end systolic ratio of noncompacted to compacted layers was greater than 2. The right ventricle appeared to be more heavily trabeculated than normal while color Doppler displayed flow within the deep intertrabecular recesses (Figure 2). These findings were consistent with the diagnosis of ventricular noncompaction. 

The patient's cardiac status continued to worsen and medical therapy was intensified. Epinephrine was added to the dobutamine infusion and hemofiltration was initiated. It was decided to place the patient on urgent priority heart transplant list. Subsequently, the patient underwent a heart transplantation three days after admission. Extracorporeal circulatory support was necessary for 24 hours post-operatively. The patient was extubated on postoperative day 6. He was discharged from the intensive care unit 17 days post-transplantation and he remained well for the subsequent 2 months.

## 3. Discussion

Noncompaction of the ventricular myocardium (sometimes referred as “spongy myocardium”) is a rare cardiomyopathy recently categorized as a primary genetic cardiomyopathy [3]. This abnormality is believed to represent an arrest in endomyocardial morphogenesis. The anatomical aspect is characterized by persistent prominent ventricular trabeculations and deep intertrabecular recesses communicating with the ventricular cavity [1]. Men appear to be affected more often than women with male patients accounting for 56–82% of cases [4]. In the largest series of patients with isolated noncompaction ventricles, the prevalence was 0.014% [5].

The disease involves the left ventricular myocardium, but right ventricular involvement is not uncommon [6]. Noncompaction of the ventricular myocardium may be isolated, but it can often coexist various cardiac or extra-cardiac anomalies, as in our case (bicuspid aortic valve). Literature reports that NVM with bicuspid aortic valve and aortic regurgitation seems to have a poor prognosis [7]. A pathological study of 14 cases of infants with NVM and unexpected death has reported a high incidence of valvular anomalies (5/14 cases, 35%) [6]. 

The clinical manifestations include systolic and diastolic dysfunction associated with heart failure signs, ventricular arrhythmias and cardioembolic events [4]. Symptoms vary among patients, ranging from asymptomatic left ventricular dysfunction to terminal heart failure. The prognosis of symptomatic patients is poor. In a series published by Ritter et al., mortality in adults during a 6 year follow-up period after onset of symptoms exceeded 47% [8]. The common causes of death are intractable heart failure or sudden cardiac death due to ventricular arrythmias. 

Diagnosis can be made by two dimension and color Doppler echocardiography. Four morphological criteria have been defined: (1) absence of coexisting cardiac abnormalities (to define an isolated NVM), (2) maximal end systolic ratio of noncompacted to compacted layers superior to 2, (3) predominant localisation of the pathology to mid-lateral, apical, and mid-inferior areas, (4) colour Doppler evidence of deep perfused intertrabecular recesses [9]. Magnetic resonance imaging, computed tomography and ventriculography can also be utilized [4]. 

Unfortunately, as demonstrated in our case, NVM is often misdiagnosed since it is not a prevalent disease. Frequently, during routine echocardiographic evaluation, the disease is identified as an incidental finding, which leads to the diagnosis being made several months to years after the onset of symptoms. Szymanski et al. published a case of NVM revealed by a ventricular arrhythmia; such arrhythmias can justify implantation of a cardioverter-defibrillator. This patient diagnosed with idiopathic cardiomyopathy, has been managed for many years with nonspecific treatment [10]. 

The management of NVM is not specific. Anticoagulation is warranted in presence of thromboembolic complications [11]. Standard medical therapy for ventricular dysfunction does not differ from other form of chronic heart failure. As in our case, cardiac transplantation has to be considered for those with refractory heart failure, although it is not a frequent therapeutic eventuality, from 4 to 12% of patients of published series [2, 4]. According to our data only 8 adult patients with NVM have been reported to have undergone heart transplantation.

## 4. Conclusion

Noncompaction of the ventricular myocardium is a rare disease often unrecognized because not widely known. Nevertheless, this cardiopathy can be accompanied by three major cardiac risks: heart failure, ventricular arrhythmias, and endocardial clot with systemic embolization. Our case report emphasizes the need for practitioner to recognize this disease not to miss a potential life-threatening pathology.
